# A Good Night’s Sleep in Malta in 2023: A Cross-sectional Study Exploring Sleep Quality and its Determinants via Social Media

**DOI:** 10.34172/jrhs.2024.137

**Published:** 2024-03-18

**Authors:** Elizabeth Grech, Sarah Cuschieri

**Affiliations:** ^1^Mater Dei Hospital, Msida, Malta; ^2^Faculty of Medicine and Surgery, University of Malta, Msida, Malta; ^3^Department of Epidemiology and Biostatistics, Western University, London, Canada

**Keywords:** Sleep, Sleep habits, Chronic diseases, Malta, Population health

## Abstract

**Background:** Sleep quality is affected by a plethora of different factors, although its relationship with chronic diseases is still unclear. This study explored perceived sleep quality and its associated determinants among the adult population of Malta.

**Study Design:** A cross-sectional study.

**Methods:** An anonymous online survey was distributed through social media targeting adults residing in Malta. Data pertaining to socio-demographic, medical history, lifestyle, well-being, sleep, and daytime sleepiness were gathered, and descriptive, univariant, and multiple binary logistic regression modelling analyses were performed.

**Results:** A total of 855 adults responded, out of whom 35.09% (95% confidence interval [CI]: 31.90, 38.41) reported sleep difficulties, especially females (81.33%; 95% CI: 76.36, 85.49), while 65.33% (95% CI: 59.61, 70.65) reported suffering from chronic disease(s). Sleep problems were positively associated with multimorbidity (odds ratio [OR]: 2.17; 95% CI: 1.38, 3.40; *P*=0.001), sleeping<6 hours (OR: 3.79; 95% CI: 1.54, 9.30; *P*=0.040), and the presence of moderate anxiety symptoms (OR: 1.99; 95% CI: 1.10, 3.59; *P*=0.020). They were also related to the presence of mild (OR: 2.25; 95% CI: 1.46, 3.45; *P*=0.001), moderate (OR: 2.40; 95% CI: 1.24, 4.64; *P*=0.010), and moderately severe (OR: 15.35; 95% CI: 4.54, 31.86; *P*=0.001) depressive symptoms after adjusting for confounders.

**Conclusion:** Chronic conditions, including anxiety and depression, along with short sleep duration, appear to contribute to poor sleep quality in Malta. A multifaceted approach is required to deal with the issue holistically and safeguard the health of current and future generations.

## Background

 The significance of sleep quality has been established as an important contributor to health, yet it is often overlooked.^1^ The quality of sleep impacts several dimensions of health, as sleep deprivation leads to aberrations in molecular, immune, and neural functions.^2^ Sleep length and quality have also been associated with mortality.^3,4^ Although it is not yet clear whether sleep issues are the result of chronic disease or whether sleep contributes to the development of chronic disease, a bidirectional relationship is plausible.^5^ The relationship between sleep and quality of life is well known, so much so that sleep quality indices have been calculated to quantify this phenomenon.^6^

 Various factors affect sleep quality, and these vary according to population demographics, occupation, behaviour, health status, and numerous other elements. For instance, sleep in children is dictated by factors such as sleep hygiene, genetics, screen time, general health, caregiver factors, and the child’s home environment.^7^ College students’ sleep quality, on the other hand, is affected by factors such as social relationships, physical activity, stress, and caffeine intake.^8^ Occupations based on rotating shifts, such as medical professions, have detrimental effects on sleep quality and circadian rhythms, and this has recently become an area of interest in modern research.^9,10^

 Poor sleep has been linked to increased cardiovascular risk through dyslipidemia.^11^ Of note, ischaemic heart disease and stroke are the top two global causes of death estimated by the World Health Organisation (WHO).^12^ These two diseases were also the leading causes of death in Malta in 2021.^13^ Malta is a small European island state found in the middle of the Mediterranean Sea and is a known cardio-metabolic country.^14^ Considering the small population size of Malta and the corresponding metabolic status, it provides the perfect landscape to study and understand sleep quality and its negative effects on the population’s health.

 This study evaluated perceived sleep quality and its associated determinants among the adult population of Malta. To the best of our knowledge, this is the first cross-sectional study to be conducted in Malta that explores these issues through social media. This study’s intention is to provide timely evidence to start the conversation pertaining to the role of sleep among cardio-metabolic populations while considering the COVID-19 pandemic’s potential effect, which is of importance in strategic planning for the management of affected individuals at primary and tertiary health care levels. Considering the cultural diversity and geographical location of Malta, this evidence can act as a framework for neighbouring countries and can be used for cross-country comparisons.

## Methods

 A cross-sectional study was performed using an online anonymous literature-based questionnaire as the tool of measure through Google Forms. The survey was disseminated between January and February 2023 through social media (Facebook®), which is considered an appropriate platform since it is mostly used to share public opinion and its users represent a substantial proportion of the global population.^15^ In fact, it was reported that around 354 000 Facebook users were registered in Malta, which contributes to a substantial proportion of the adult population (20 + years total population of 425 382).^16,17^ Notably, social media users do not only include individuals but also businesses, associations, groups, and potentially individuals with duplicate accounts.

 Anyone living in Malta aged 18 + years was included in the study; this was clearly indicated within the social media post uploaded by the authors and was part of the survey’s introductory page before the participant gained access to the questionnaire. Participants were informed that their opting to participate in the survey was considered informed consent.

###  Data collection

 The questionnaire was divided into five sub-sections. The sociodemographic and medical history sections followed the European Health Information Survey.^18^ The sleep health and lifestyle sections were adopted from the study by Ruiz-Castell et al and included the Epworth Sleepiness Scale.^5,19^ Those who reported having sleep difficulties were asked whether these difficulties were related to the onset of COVID-19 or the acquisition of COVID-19. The wellbeing section featured the ‘General Anxiety Disorder 7’ (GAD-7) score and the ‘Patient Health Questionnaire – 9’ (PHQ-9) score.^20,21^

###  Definitions

 The residential districts followed the Eurostat System of Local Administrative Units. The six districts of Malta and Gozo are Northern Harbour, Southern Harbour, Southeast, Western, Northern, and Gozo.^22^ Multimorbidity was defined as the co-occurrence of at least two chronic diseases.^23^ Sleep quality was defined as participants’ self-perceived sleeping difficulties. The Epworth scale was used to measure daytime sleepiness.^19^ Experiencing anxiety and depression symptoms over two weeks were assessed through the GAD-7 and PHQ-9 scores.^21,24^

###  Data analysis

 The GAD-7 score was measured by following the method and the cut-off point reported by Spitzer et al^20^ Participants had to respond to seven questions targeting different aspects of anxiety, and a score was calculated as (*i*) not at all (score 0), (*ii*) several days (score 1), (*iii*) more than half the days (score 2), and (iv) nearly every day (score 3). The total score was then determined and categorized as (*i*) < 5 as normal, (ii) 5–10 as mid-anxiety, (*iii*) 10–14 as moderate anxiety, and (*iv*) 15 + as severe anxiety.

 The PHQ-9 score was measured by following the method and the cut-off point suggested by Kroenke et al^21^ Participants were asked to respond to a nine-item depression subscale, with a score associated with each response selection as (*i*) not at all (score 0), (*ii*) several days (score 1), (*iii*) more than half the days (score 2), and (*iv*) nearly every day (score 3). The total score was then computed and classified as (*i*) 5–9 as mild depression, (*ii*) 10–14 as moderate depression, (iii) 15–19 as moderately severe depression, and (*iv*) 20 + as severe depression.

 The Epworth Sleepiness Score was measured following the method and the cut-off point presented by Johns in 1991.^19^ This score assessed the chance of dozing off in eight different situations, with a score associated with each response selection as (*i*) would never doze (score 9), (*ii*) a slight chance of dozing (score 1), (*iii*) a moderate chance of dozing (score 2), and (*iii*) a high chance of dozing (score 3). The total score was then estimated, and a score of 11 or more was considered to experience “sleepiness”.^5^

###  Statistical data analysis

 Frequencies were used to describe study population characteristics and sleep quality. A categorical analysis through a chi-square or Fisher’s test was performed between those reported experiencing “sleeping problems” and different variables. The variables, found to be significant following categorical analyses, were then utilized for univariant analyses through a binary logistic regression. Significant categorical variables identified through univariant analyses were then considered confounding variables for the multiple regression and added to the model following main effect modelling. The dependent variable was taken as “having sleeping problems”, with the reference being “no sleeping problems”. Analyses were performed using IBM SPSS (version 21) for Mac, and a *P *value less than 0.05 was considered to be statistically significant.

## Results

 A total of 855 adults out of an estimated 354 000 Facebook users in Malta^16^ responded to the online survey with a female predominance (Male: 23.04%, n = 197; Female: 76.84%, n = 657; Others: 0.12%, n = 1). Most responders were middle-aged (59.42%; 95% CI: 56.03, 62.71), resided within the Northern Harbour district, the area with the highest population density in Malta (34.74%; 95% CI: 31.56–38.05), were employed (75.68%; 95% CI: 72.62, 78.50), and were married (67.96; 95% CI: 64.69, 71.07). The majority of participants were nonsmokers (65.85%; 95% CI: 62.55, 69.01), performed minimal physical activity if at all (78.71%; 95% CI: 75.78, 81.38), and consumed four or less vegetables and fruit daily (92.16%; 95% CI: 90.10, 93.83).

 More than half of the study participants (55.56%; 95% CI: 52.15, 58.91) reported a medical history of at least one chronic disease. Table 1 provides an overview of different chronic diseases reported by participants, while Table 2 presents participant demographics as well as different variables, including the frequency of having one chronic disease or multimorbidity and the categorized GAD-7 and PHQ-9 scores among the study population. Hypertension (14.15%; 95% CI: 11.92, 16.71) and mental health disorders (10.41%; 95% CI: 8.48, 12.70) were the most common medical issues, while multimorbidity was present in less than a quarter (23.27%; 95% CI: 20.51, 26.28) of the study population.

**Table 1:** Medical history reported by participants

**Health-related characteristics **	**Number**	**Percent**
Heart problems	22	2.57
Hypertension	121	14.15
Stroke	2	0.23
Circulation problems	5	0.58
Respiratory problems	68	7.95
Diabetes mellitus	42	4.91
Cirrhosis	1	0.12
Urinary incontinence	8	0.94
Kidney problems	2	0.23
Mental health	89	10.41
Fibromyalgia	27	3.16
Cancer	11	1.29
Stomach problems	71	8.3
Obesity	71	8.3
Dyslipidemia	62	7.25
Chronic back or neck disorder	60	7.02
Rheumatoid arthritis	14	1.64
None	380	44.44

**Table 2:** Sleep Difficulties and Associated Variables

**Health-related characteristics**	**Difficulty sleeping**			**Univariant analyses**		**Multivariable model**	
	**Yes**	**No**	***P *** **value**	**OR (95% CI)**	***P *** **value**	**OR (95% CI)**	***P *** **value**
Gender							
Male	56	141	0.030	0.55 (0.45, 0.94)	0.023	-	0.073
Female	244	413		Ref.			
Age groups (year)							
18-19	3	11	0.100	-	0.894	-	-
20-29	25	61		-	0.700	-	-
30-39	51	110		0.77 (0.54, 0.87)	0.052	-	0.333
40-49	86	183		0.45 (0.30, 0.60)	0.010	-	0.346
50-59	98	141		0.66 (0.50, 0.88)	0.021	-	0.208
60-69	30	42		-	0.060		
70-79	6	7		Ref.			
District							
Southern harbour	34	77	0.051	-	0.824	-	
Northern harbour	109	188		1.41 (1.00, 2.66)	0.041	-	0.272
South eastern	29	53		-	0.250	-	
Western	57	103		1.09 (1.01, 2.47)	0.053	-	0.373
Northern	60	107		1.41 (1.05, 2.04)	0.050	-	0.159
Gozo	11	27		Ref.			
Education							
Up to secondary	41	74	0.040	-	0.620	-	0.343
Up to sixth form	51	95		-	0.874	-	
Undergraduate degree	103	199		0.85 (0.44, 0.98)	0.050	-	
Post-graduate degree	105	187		Ref.			
Employment							
Employed	220	421	0.020	1.36 (1.12, 8.45)	0.030	-	0.163
Student	16	42		-	0.362	-	
Retired	24	29		-	0.870	-	
Stay at home	36	43		-	0.611	-	
Self-employed	2	14		Ref.			
Living status							
Single, live alone	16	26	0.050	-	0.982	-	
Single, live with someone else	47	102		0.85 (0.03, 0.99)	0.051	-	0.306
Married	199	378		1.36 (1.10, 3.65)	0.050	-	0.470
Separated, live alone	12	16		-	0.740	-	
Separated, live with someone else	15	16		-	0.690	-	
Divorced, live alone	2	4		-	0.410	-	
Divorced, live with someone else	2	6		-	0.372	-	
Widowed, live alone	3	2		-	0.650	-	
Widowed, live with someone else	2	1		Ref.			
Smoking status							
No	194	369	0.044	Ref.			0.177
Yes	65	107		1.13 (1.03, 5.02)	0.012	-	
Ex-smoker	41	79		-	0.543	-	
Physical activity (hours/week)							
Never	122	209	0.050	Ref.			0.940
< 3	118	224		1.28 (1.01, 1.74)	0.030	-	
> 3	60	122		-	0.744	-	
Vegetables and fruit daily (portion /day)							
< 1	105	208	0.042	-	0.801		
1-4	172	303		-	0.783		
≥ 5	23	44		Ref.			
Multimorbidity							
No	104	289	0.001	Ref.			
One chronic disease	96	167		2.67 (1.86, 3.82)	0.010	-	0.084
Multimorbidity	100	99		1.60 (1.14, 2.25)	0.001	2.17 (1.38, 3.40)	0.001
GAD-7 score						-	-
No	134	341	0.001	Ref.			
Mild	85	144		1.52 (1.09, 2.13)	0.020	-	0.364
Moderate	49	47		2.70 (1.72, 4.24)	0.001	1.99 (1.1, 3.59)	0.020
Severe	32	23		3.42 (1.92, 6.08)	0.001	-	0.180
PHQ-9 score							
No	150	408	0.001	Ref.			0.001 0.010 0.001 0.097
Mild depression	83	105		2.16 (1.53, 3.06)	0.001	2.25 (1.46, 3.45)	
Moderate depression	35	32		2.94 (1.76, 4.93)	0.001	2.40 (1.24, 4.64)	
Moderately severe depression	24	7		10.32 (4.12, 25.83)	0.001	15.35 (4.54, 31.86)	
Severe depression	8	3		6.28 (1.60, 24.60)	0.001	-	
Hours slept at night (not working the next day)							
< 6	93	61	0.001	6.13 (1.23, 10.15)	0.011	3.79 (1.54, 9.30)	0.040
6-9	195	440		-	0.640	-	-
> 9	12	54		Ref.			
Hours slept at night (working the next day)							
< 6	160	135	0.001	2.44 (1.33, 8.36)	0.040	-	0.294
6-9	139	415		2.99 (1.33, 5.36)	0.030	-	0.876
> 9	1	5		Ref.			
Epworth score			0.050	Ref.			
Normal ( < 11)	277	507		1.36 (1.06, 9.32)	0.052	0.46 (0.24, 0.87)	0.020
Sleepiness ( ≥ 11)	23	48					

*Note*. CI: Confidence interval; GAD-7: General Anxiety Disorder 7; PHQ-9: Patient Health Questionnaire – 9.

###  Sleep quality

 Out of the total study population, 35.09% (95% CI: 31.90, 38.41; n = 300) reported to have sleep difficulties, with a female predominance (81.33%; 95% CI: 76.36, 85.49; *P*= 0.030), and 16.67% (95% CI: 12.73, 21.48; n = 50) claimed that their doctor diagnosed them with a sleep disorder. The majority of them reported that their sleeping difficulty was present before the onset of COVID-19 (85.67%; 95% CI: 81.07, 89.33). Approximately 12.67% (95% CI: 9.23, 17.09) and 1.67% (95% CI: 0.62, 4.07) developed a sleeping disorder following the onset of COVID-19 and after the acquisition of the COVID-19 infection, respectively.

Table 2 provides sleep quality characteristics among the study population. On categorizing the Epworth score in accordance with the reported sleep difficulties, less than one-tenth were identified as experiencing daytime sleepiness. Most of the participants reporting sleep difficulties claimed to sleep less than six hours when they were to be working the next day, although sleep duration appears to be longer for most when they are not working the following day (Table 2). Those with sleep difficulties reported a higher frequency of chronic diseases (65.33%; 95% CI: 59.61, 70.65) than those without such difficulties (47.93%; 95% CI: 43.71, 52.17, *P*< 0.001), as well as higher GAD-7 (*P*< 0.001) and PHQ-9 (*P* < 0.001) scores (Table 2).

 Following multiple binary logistic regression modelling, sleep problems were positively associated with the presence of multimorbidity (OR: 2.17; 95% CI: 1.38, 3.40; *P*= 0.001), sleeping less than 6 hours if one does not have work the next day (OR: 3.79; 95% CI: 1.54, 9.30; *P*= 0.040), and with the presence of moderate anxiety symptoms (OR: 1.99; 95% CI: 1.1, 3.59; *P*= 0.020). They were also attributed to the presence of mild (OR: 2.25; 95% CI: 1.46, 3.45; *P*< 0.001), moderate (OR: 2.40; 95% CI: 1.24, 4.64; *P*= 0.010), and moderately severe depressive symptoms (OR: 15.35; 95% CI: 4.54, 31.86; *P*< 0.001) after adjusting for gender, residing districts, education, employment status, living status, smoking habits, and physical activity.

## Discussion

 The long-term consequences and effects of long-term sleep deprivation and poor sleep health have been well documented. There is an association with the development of obesity, hypertension, cerebrovascular and cardiovascular events, diabetes, and mental health disorders.^25^ This study thus set out to establish perceived sleep quality and its associated determinants among the adult population of Malta, a known cardio-metabolic population.^26^ The percentage distribution of the respondents in this study mirrors the population distribution of residents within the six districts, including the Southern Harbour study population (13.00%) vs. the national population (16.45%), and the Northern Harbour study population (34.78%) vs. the national population (33.25%). The population distribution of the other districts included South Eastern study population (9.60%) vs. national population (14.50%), Western study population (18.74%) vs. national population (12.19%), Northern study population (19.56%) vs. national population (16.91%), and finally, Gozo study population (4.45%) vs. national population (6.70%). Although this does not make the study nationally representative, it does imply that the collected data are reflective of population distribution in terms of districts. The majority of study participants were middle-aged (40‒60). This age group constitutes a large proportion of the working population and will be the next generation to retire.^27,28^ The sleep health of middle-aged individuals is important, as sleep affects many aspects of current and future health and productivity.^29^ Once this population retires, the burden on health systems will also be mitigated if the health of workers is safeguarded early on, and this will also have a positive impact on the economy.^30,31^

 Although studies have shown that COVID-19 has led to sleep disruption, the majority of our study population stated that their sleep issues started before the onset of COVID-19.^32^ Malta is a hub for metabolic diseases such as diabetes and obesity, and these chronic illnesses themselves may contribute to poor sleep and be the possible causes of our study participants’ poor sleep health.^33,34^ Conversely, poor sleep health has been associated with a higher body mass index and a greater risk of developing diabetes.^35^ This may further contribute to the development of chronic metabolic diseases in Malta. Indeed, participants reporting sleep problems were more likely to suffer from a chronic disease and experience anxiety and depressive symptoms. Although it has not yet been established whether issues with sleep are a result of chronic disease or whether sleep contributes to the development of chronic disease, a bidirectional relationship is possible.^5^

 The Epworth Sleepiness Scale measures daytime sleepiness. Our results demonstrated a negative association between a high Epworth score and having sleep problems after adjusting for the same cofounders. This could be the result of a small sample size or may be due to other confounding factors affecting this relationship. It could also imply that the sleep problems in Malta arise from other causes, such as chronic diseases that do not contribute to daytime sleepiness but may result in shorter periods of sleep, a factor known to contribute to the development of chronic diseases and potentially to multimorbidity.^36^

 Multimorbid patients, meaning those with two or more chronic diseases, were twice as likely to experience sleep problems and had moderate anxiety and depressive symptoms. These issues further exemplify a relationship between all these factors and poor sleep quality. Further research is required to determine potential causative factors and repercussions, strengthening the association between chronic diseases.

 The majority of participants who reported having sleep difficulties claimed to sleep less than six hours per night before going to work the next day. Work-related stress has been associated with short sleep duration, meaning that people may get fewer hours of sleep before a day of work, potentially due to stressors pertaining to work itself.^37^ Although Malta is a small island (316 km^2^), traffic congestion and a lack of parking are major issues in islanders’ everyday lives.^38,39^ The most popular method of transportation is private vehicles.^40^ All these issues indicate that residents may have to sacrifice sleep in order to leave home in time to beat traffic and park their cars. Indeed, this was one of the potential reasons contributing to spatial health inequalities between mainland Malta and its sister island, Gozo.^25^

 Noise pollution is another nationwide issue that may contribute to poor sleep health.^41^ Concerns pertaining to noise pollution in Malta and its effects on health have recently captured the media’s attention.^42,43^ Construction is rampant on the island and generates noise for long periods of time during the day.^44^ This has led to residents speaking out and raising issues such as not being able to study for exams and having high levels of stress and frustration as a result of the noise.^43^ Another source of noise pollution is music coming from bars, nightclubs, and restaurants, which is sometimes heard through to the early morning.^45^ All these factors have an impact on sleep quality and are widespread enough to affect a good proportion of the Maltese population, which may have contributed to our results.^46^

 Given that the study was performed using social media, it cannot be mentioned that it is representative of the whole adult population of Malta. It does, however, provide a good indication of the sleep quality of adults in Malta, However, it represents that people who did not have Facebook® at the time of the study were excluded from participating in the study. This may have included a significant proportion of the elderly population and the digitally illiterate. Future studies might consider employing bootstrapping and cross-validation to mitigate overfitting, and optimism models should be developed in this regard.

 Study responses may also have been subject to self-reporting bias, where participants may not have been entirely truthful, and recall bias, where respondents may have misremembered certain facts and thus answered questions inaccurately. These are the limitations of any survey. The small sample size itself limited the study given that participation was voluntary and was based on participants’ willingness to answer the questionnaire. This may have led to type 2 errors (false negatives), as the small sample size may have failed to detect associations between sleep quality and contributing factors. The power of the study might have been affected as well. Given that this is a cross-sectional study rather than a longitudinal study, it was impossible to determine the causality of associations.

**Highlights**

- This study explored perceived sleep quality and its associated determinants.
- The effects of sleep quality on chronic diseases are still unclear.
- Chronic conditions appear to contribute to poor sleep quality in Malta.
- A multifaceted approach is required to deal with sleep quality holistically.

## Conclusion

 Good sleep hygiene, duration, and quality of sleep are major contributing factors to day-to-day as well as long-term health. Although sleep quality and contributing factors have been popular topics in recent research, further studies are required to truly understand the consequences of poor sleep on health at the population level. The authors hope that this study will prompt further investigation into an often-overlooked contributing factor to metabolic disease. Chronic conditions such as obesity, diabetes, and hypertension are global issues. They may be the result of poor sleep or potentially contribute to poor sleep quality. A multifaceted approach is required to deal with the issue holistically to safeguard the health of current and future populations.

## Authors’ Contribution

**Conceptualization:** Sarah Cuschieri.

**Data curation:** Sarah Cuschieri, Elizabeth Grech.

**Formal analysis:** Sarah Cuschieri.

**Investigation:** Sarah Cuschieri, Elizabeth Grech.

**Methodology:** Sarah Cuschieri, Elizabeth Grech.

**Project administration:** Sarah Cuschieri, Elizabeth Grech.

**Resources:** Sarah Cuschieri, Elizabeth Grech.

**Software:** Sarah Cuschieri, Elizabeth Grech.

**Supervision:** Sarah Cuschieri.

**Validation:** Sarah Cuschieri, Elizabeth Grech.

**Visualization:** Sarah Cuschieri, Elizabeth Grech.

**Writing–original draft:** Sarah Cuschieri, Elizabeth Grech.

**Writing–review & editing:** Sarah Cuschieri, Elizabeth Grech.

## Competing Interests

 None.

## Ethical Approval

 Ethical approval was granted by the Research Ethical Committee, University of Malta (MED-2023-00052).

## Funding

 This research received no specific grant from funding agencies in the public, commercial, or not-for-profit sectors.
